# The Telomere/Telomerase System in Chronic Inflammatory Diseases. Cause or Effect?

**DOI:** 10.3390/genes7090060

**Published:** 2016-09-03

**Authors:** Vasileios Kordinas, Anastasios Ioannidis, Stylianos Chatzipanagiotou

**Affiliations:** 1 Third Department of Internal Medicine and Diabetes Centre, Saint Panteleimon General Hospital, Nikea-Pireaus 18454, Greece; 2 Department of Clinical Microbiology and Medical Biopathology, Athens Medical School, Aeginition Hospital, Athens 11528, Greece; tasobi@uop.gr (A.I.); schatzi@med.uoa.gr (S.C.); 3 Department of Nursing, Faculty of Human Movement and Quality of Life Sciences, University of Peloponnese, Sparta 23100, Greece

**Keywords:** telomerase, telomeres, inflammation, senescence

## Abstract

Telomeres are specialized nucleoprotein structures located at the end of linear chromosomes and telomerase is the enzyme responsible for telomere elongation. Telomerase activity is a key component of many cancer cells responsible for rapid cell division but it has also been found by many laboratories around the world that telomere/telomerase biology is dysfunctional in many other chronic conditions as well. These conditions are characterized by chronic inflammation, a situation mostly overlooked by physicians regarding patient treatment. Among others, these conditions include diabetes, renal failure, chronic obstructive pulmonary disease, etc. Since researchers have in many cases identified the association between telomerase and inflammation but there are still many missing links regarding this correlation, the latest findings about this phenomenon will be discussed by reviewing the literature. Our focus will be describing telomere/telomerase status in chronic diseases under the prism of inflammation, reporting molecular findings where available and proposing possible future approaches.

## 1. Telomeres, Telomerase and Inflammation: An Introduction

The ends of linear chromosomes are complex heterochromatin structures containing multiple distinct protein elements [1,2]. This particular motif serves to protect the chromosome and preserves the stability of the genome [3,4]. After each cell division, a small part of this far-end sequence is lost and when telomeres reach a critical length, the cell either dies through programmed cell death, known to us as apoptosis or it enters a state of permanent cell cycle arrest called replicative/cellular senescence [5,6,7]. Many researchers have very accurately characterized telomeres as the molecular clock of the cells [8,9]. Telomeres are elongated by the actions of an enzyme called telomerase that contains many proteins, but two of its components seem to be essential for its action [10,11]. These are the RNA component, serving as a template to the telomeric sequence, and the catalytic subunit, a reverse transcriptase synthesizing new telomeres having its own RNA as a template [12,13]. This way the cell can compensate for telomeric loss continuing its divisions [14,15]. However, since immortality is not a human trait, telomerase is not active in the majority of human mature cells and that is why most of the somatic cells eventually die, a phenomenon directly responsible for ageing [16,17,18,19]. The enzyme is active during gestation, in immature undifferentiated cells, and in very few mature cells such as lymphocytes [16,17,18].

Diabetes, chronic obstructive pulmonary disease, renal failure and other chronic diseases have as a common characteristic the increased synthesis of pro-inflammatory cytokines and the disruption of the proper association/balance between pro-inflammatory and anti-inflammatory cytokines [20,21,22]. Interferon-γ (IFN-γ), tumor necrosis factor a (TNF-a), interleukin 1 and 6 (IL-1, IL-6) are only a handful of the molecules that have been found to be elevated in these conditions either in high or in low grade inflammatory levels [23,24,25]. Chronic inflammation is extensively studied because it has been found to be responsible for disease progression, poor outcome of therapy, poor quality of life and as a result of the above health care cost increase [20,21,22,26]. In addition, many studies have managed to link chronic inflammation with telomere/telomerase biology impairment, a phenomenon contributing among others to immune system impairment of these patients [20,27]. Whether inflammation is triggering telomerase/telomere dysfunction or vice versa still remains to be elucidated [20,28,29,30].

Since telomerase plays an extremely important part in cancer and since this association is studied by a plethora of laboratories around the world, it is deliberately chosen not to include the telomerase and cancer connection in this manuscript [31,32]. During this study, we will analyze the most common chronic conditions, as well as habits that affect public health like smoking, diet and alcoholism in an effort to describe how telomerase biology and chronic inflammation are associated.

Acute inflammation is a protective response to injury of a process that delivers leukocytes and plasma proteins like antibodies, to sites of infection or tissue damage while it also induces neutrophilic infiltration [33]. Acute inflammation lasts usually for hours and has many positive (e.g., IL-6/TNF-a) and negative (e.g., IL-10) regulators [34]. If infection and/or tissue damage persist, or if the healing process is somehow disturbed, or if one of the negative control mechanisms fails, inflammation may progress to a chronic state that can last for weeks, months or maybe years [33,34]. In many common chronic diseases, chronic inflammation probably does not follow a manifestation of an acute reaction but it most likely begins as a low grade, smoldering response contributing to disease initiation and progression [33].

The network of pro- and anti-inflammatory stimuli originating in the beginning of inflammation is a very complex and tightly organized one [34,35,36,37]. The most well studied inflammation related molecules are interleukin 1 (IL-1) and TNF receptor families and also the Toll-like microbial pattern recognition receptors (TLRs) which also belong to the IL-1R family [34,35,38,39]. IL-1 and TNFa are pro-inflammatory cytokines which are rapidly released upon injury or infection [34,35]. TLRs on the other hand recognize microbial molecular patterns and that is why they are called pattern recognition receptors (PRR) [34,35,40]. Many scientists suggest that apart from pathogens, endogenous ligands may as well trigger TLRs during certain disease states, a phenomenon that can possibly act to promote inflammation in the absence of infection [34,35]. One of the next steps in the inflammatory process is the recruitment of further signaling proteins that belong to the TRAF family and various protein kinases, like IRAK1 and IRAK4 as well as RIP kinases which activate many molecular pathways, the most important being possibly the activation of Mitogen Activated Protein Kinases (MAPK) including JNK and p38 MAPK, as well as IκB kinases (IKK) [34,35,41]. MAPKs are responsible for transcriptional regulation of many pro-inflammatory genes and lead to the activation of the transcription factor NF-κΒ, a central regulator of inflammation and immunity. As a result, many pro-inflammatory mediators are expressed in order for inflammation to be initiated and maintained [34,35,42].

All the above processes are also subject to negative regulation in order for inflammation to be resolved. In fact, many pro-inflammation pathways trigger the expression of certain anti-inflammatory molecules such as IL-10 [34,35,43]. Many phosphatases like PTEN phosphatase (PTP family) and MAP kinase phosphatases (MKPs) are major negative controls for inflammation while, at the same time, certain ubiquitin ligases function as inhibitory molecules for the inflammatory procedure [34,35,44]. Such an ubiquitin ligase is A20, a direct target gene for the NF-κB pathway and its action result in the downregulation of NF-κB activity [34,35,45]. Adaptor molecules TRAF3 and TRAF6 are also worth mentioning. Studies have shown that the balance between the signals generated by these two proteins play essential roles in controlling and properly resolving inflammation [34,35,46]. The above are just a few among many mechanisms involved in halting inflammation. Studies have shown that deficiency in just one negative regulator can trigger serious inflammatory disorders while the disruption of any of these molecular pathways can result in a chronic inflammatory status [34,35].

Aging is a natural process characterized by telomeric attrition and chronic inflammation and telomeres are the emerging marker of health and age related disease [20]. Similar to an aged organism, many chronic disorders also exhibit signs of accelerated or premature aging [47,48]. However, what is exactly the connection between telomeres and inflammation? Do short telomeres trigger an inflammatory status or does the deregulated immune system cause telomere/telomerase deficiency? The answer is probably both. As said above, after certain divisions, telomeres reach a critical length that eventually leads to apoptosis or replicative senescence. Senescence is a state of permanent inhibition of cell proliferation and it has been observed in many tissues and cell types both in vivo and in vitro [49,50]. Two types of senescence have been described, telomere dependent and stress induced premature senescence (SIPS) [51,52]. Senescent cells exhibit short telomeres, are resistant to apoptosis and have no telomerase activity [53,54]. Moreover, senescence causes a loss in tissue repair/regeneration capacity while cells undergoing senescence are metabolically active producing increased concentrations of proinflammatory cytokines like TNF-a, IL-6 and overactivation of NF-κB [20,51,53]. Telomere attrition is considered to be such a strong senescence inducing factor that even one dysfunctional telomere can cause permanent cell cycle arrest [20]. Moreover, the loss of telomeric sequence is among others a signal that can stimulate the production of many pro-inflammatory cytokines by different cell types during senescence, such as cultured fibroblasts and endothelial cells, a phenomenon called “senescence associated secretory phenotype” (SASP) [20,53,55]. Cells during this state have the ability to produce molecules like cytokines, chemokines, growth factors and proteases, but the exact secretion profile varies between cells and induction stimuli [20,53,56,57]. These secreted factors are involved in the maintenance of senescence, alter the micro-environment, chemo-attract immune cells and promote tumorigenesis [20,55]. Though senescence seems to be a cancer protective mechanism, through SASP this procedure probably contributes to aging and age related diseases by producing a low grade inflammation state [58,59]. For example, evidence show that senescent vascular cells promote atherosclerosis, while senescent adipocytes can lead to insulin resistance [51,52,60]. The above data suggest that cells undergoing SASP produce molecules that may cause local inflammation which in time can turn into a chronic condition [20,52,53,56,57].

Many chronic conditions exhibit signs of premature aging and as stated above telomere dysfunction seems to be an important aspect of chronic inflammation which is observed during the aging process [20,53]. On the other hand, although the data are still inconclusive, there are many indications that chronic inflammation can also cause telomere/telomerase dysfunction directly [61]. For example, there are many proteins participating in molecular pathways both in telomerase expression and in the inflammatory process [6,20,53]. NF-κB has been shown to regulate telomerase expression apart from its properties in promoting inflammation whereas RAP1 a protein involved in telomeric lengthening is also involved in NF-κB signaling pathways [20,62]. Moreover, reactive oxygen species (ROS) produced during inflammation can affect telomeric DNA, trigger DNA damage response pathways (DDR) and finally cause cellular senescence [20,63,64]. ROS have been observed to exhibit an increase in their production due to dysfunctional mitochondria, a common phenomenon during accelerated aging diseases [20,63]. In addition, ROS have been found to contribute in NF-κB overexpression during chronic inflammatory states [65]. Moreover, Jurk et al. reported that low grade chronic inflammation in mice can directly cause ROS mediated telomeric DNA damage, and that this dysfunction can be rescued via anti-inflammatory therapy [20,53,61,63]. Additionally, apart from telomere shortening, a state of senescence can also be reached by direct DNA damage [66,67]. Evidence suggests that telomeres are important targets of stress, accumulating DNA damage foci and causing a prolonged DDR during cellular senescence [66,67]. In other words, it seems that telomeric DNA is more sensitive to damage irrespectively of telomere length or telomerase activity. If telomeres become damaged, they become irreparable and trigger persistent DDR and cellular senescence [66,67].

Telomeres could serve as a biological marker of aging and tissue function regarding the diagnosis of many age related diseases. A large percentage of studies have focused on analyzing leukocyte telomere length but since there exists an interindividual variability of telomeric length in different tissues, leukocyte telomere length should not be considered as the mean telomeric length of an organism as a whole [68]. Below we will discuss the telomere/telomerase status in some serious chronic inflammatory conditions and where available we will analyze how this status is connected or not with inflammation.

## 2. The Telomere/Telomerase System in Chronic Disorders. Is Inflammation to Blame?

### 2.1. Chronic Lung Diseases

Chronic obstructive pulmonary disease (COPD) and idiopathic pulmonary fibrosis (IPF) are very serious chronic disorders and major public health issues [69,70]. COPD is a lung condition associated with irreversible airflow obstruction as a consequence of small airways disease, excessive mucous production and emphysema. Smoking and inflammation have been identified as the leading causes for COPD onset and progression [69,70,71]. TNF-a among other cytokines has been found to be elevated in COPD and its concentration seems to increase even more during disease exacerbations [72]. Many scientists have proposed that COPD is a disease of accelerated aging and thus the possible relationship between COPD and telomeres/telomerase is under investigation [73]. Studies have also associated COPD with shorter telomere lengths in alveolar epithelial and endothelial cells, fibroblasts, smooth muscle cells as long as in circulating leucocytes [73,74,75]. In addition, a strong negative correlation has been observed between lung cells’ telomere length and the susceptibility to replicative cell senescence in vitro. Moreover, it has recently been reported that subjects with COPD exacerbation exhibit higher levels of copper, zinc, CRP and serum telomerase, findings associated with oxidative stress [76]. Zhou et al. discovered that SASP occurs both in vivo and in vitro in airway epithelial cells. Moreover, this senescent associated inflammatory state was positively regulated by p38 MAPK activation, a pathway identified as critical in the production of many pro-inflammatory cytokines [77]. Many believe that SASP is an important driver of chronic inflammation and, therefore, part of a vicious cycle of inflammation, DNA damage, and senescence in chronic lung diseases [75,78]. Thus, SASP may contribute to lung alterations and lung tissue remodeling. Moreover, SASP originating in the lung may propagate the senescence process not only to neighboring lung cells, but potentially to remote organs as well, inducing sustained chronic inflammation [75]. It must also be noted that mutations in telomerase reverse transcriptase gene *hTERT* have been identified in patients suffering from severe lung emphysema [75,79]. Interestingly, Amsellem et al. discovered a direct association between short telomeres, reduced telomerase activity and the overproduction of major pro-inflammatory cytokines like IL-6 and IL-8 in endothelial cells from COPD patients [75,78]. Moreover, the work by Birch et al. must also be stated since they discovered dysfunctional telomeres in airway epithelial cells from patients with COPD, which can be accelerated from smoking and may be associated with the secretion of inflammatory cytokines IL-6 and Il-8 [80]. The same group of researchers also reported dysfunctional telomeres and the activation of senescence pathways in the airways of patients with bronchiectasis [81].

Idiopathic pulmonary fibrosis (IPF) is a life-threatening lung degenerative disease that lacks current effective treatments and is characterized by a dysregulated wound healing response and the presence of lung scarring, immune infiltrates, and inflammation [82,83]. Although there is some controversy regarding the inflammation theory in IPF pathogenesis and progression, it is still widely studied since it is considered a major factor leading to end stage IPF [84]. As it was discussed previously about COPD, similar findings have been reported about telomere/telomerase dysfunction in patients with IPF. For example, leucocyte telomere shortening and *hTERT* mutations have also been reported in patients with lung fibrosis [75]. At the cellular level, IPF is characterized by alveolar epithelial injury, initiation of inflammatory cascades, exaggerated pro-fibrotic cytokine expression, increased extracellular matrix (ECM) deposition, and the development of fibrotic lesions. Although many aspects of disease initiation and progression need to be elucidated, it is possible that telomere attrition plays an important role in IPF chronic inflammation which in turn contributes to the chronic disrupted wound healing process observed in lung fibrosis [83]. Apart from telomere shortening, many telomerase mutations associated with IPF have been discovered since 10% or 15% of patients carry mutations in either telomerase reverse transcriptase gene or its RNA component (*hTR*) [83,85]. It has been proposed that short telomeres may be a risk factor for the development of IPF since even in patients without mutations in telomerase genes short telomeres seem to be a common trait among these individuals [86]. Finally, Chen et al. demonstrated that telomerase deficient mice exhibit alveolar stem cell replicative senescence, formation of alveola r sacs and a characteristic inflammatory phenotype with a remarkable elevation of various cytokines such as Il-1, Il-6, TNF-a and others [87].

One of the major causes for the lung diseases discussed earlier is smoking, which has been identified as a major public health threat having deleterious effects in the organism’s respiratory system, causing systemic oxidative stress, being responsible for carcinogenesis and inducing immune system dysfunction including inflammation [88]. The effects of smoking in the telomere/telomerase system have not yet been extensively studied and the results are in many manuscripts contradictory. It has correctly been proposed that due to oxidative stress caused by smoking, cells age faster and telomere attrition must be higher in smokers. Although some studies have reported exactly this phenomenon, others found no associations at all between smoking and telomere length, while others discovered short telomeres in smokers’ leucocytes but the telomere attrition rate was found to be slower in the long term [89,90]. Though the results from many reports are contradictory, it is believed that chronic smoke exposure affects telomere length and telomere attrition rate but the details behind this possible phenomenon still remain scarce [89,90]. On the other hand, smoking is associated with higher telomerase activity in vitro, while certain telomerase mutations have been discovered that cause severe emphysema in carriers that smoke [79,91].

### 2.2. Diabetes

Diabetes mellitus is a major public health issue, which contains a spectrum of diseases with the most important ones being type 1 diabetes (T1D) and type 2 diabetes (T2D). T1D is an autoimmune disease while T2D which accounts for approximately 90%–95% of diabetic patients has a complex disease pathogenesis [92]. There have been many reports recognizing a low grade chronic inflammation in diabetes as pathogenesis and a disease progressing mechanism [92,93]. IL-1b, IL-6, TNF-a and CRP are only a few of the inflammatory molecules found to be elevated in diabetic patients [93,94]. Diabetes is associated with inflammation and oxidative stress, while oxidative damage can also determine telomere shortening. By that notion, several studies have identified a connection between telomere/telomerase dysfunction and diabetes although there are still many aspects that need further research. Telomeres have been found to be shorter in leucocytes from diabetics while in monocytes, telomeres were not only found to be shorter but telomeric length was inversely correlated with oxidative stress [95]. In addition, telomerase activity seems to be lower in leucocytes from diabetic patients when compared to normal controls [95]. Moreover, telomeric length in peripheral blood mononuclear cells (PBMCs) is associated with the duration of disease and good glycemic control seems to be protective for telomeric loss [95,96,97].

Several mouse models have been used to examine the telomere/telomerase dynamics in diabetics. When mice are induced to exhibit high pancreatic beta cell mass, *hTERT*/telomerase expression is also higher, and thus the subjects do not develop diabetes. Accordingly, when low beta cell mass is induced, telomerase is under-expressed and mice become more susceptible in developing diabetes [95]. Interestingly telomerase null mice, exhibit normal insulin levels at basal state, but when sugar was added, blood glucose exhibited a much slower reduction rate due to lower insulin production [98,99]. It is also important to state that telomerase has been found to be upregulated in vascular smooth muscle cells of diabetic patients. This finding is crucial since high proliferation of these cells contributes to atherosclerosis and vascular disease [100].

In both T1D and T2D, leucocyte telomere length was found to be shorter than normal controls and correlated inversely with patients’ oxidative stress status [101]. In addition, telomere shortening ratio was also found inversely correlated with glycated hemoglobin, age, fasting plasma glucose and waist circumference among others [101]. Another important finding is that telomere length in patients with T1D is an important predictor factor for diabetic nephropathy progression while Astrup et al. observed that telomere length in white blood cells from T1D patients is associated with all-cause mortality [7,96,102]. It has also been observed that telomere loss in T2D patients contributes to oxidative stress and endoplasmic reticulum stress while telomere shortening has also been proposed that it can serve as an independent risk factor of T2D and it can measure disease progression [96]. Moreover, telomere shortening in T2D patients seems to involve mitochondrial dysfunction as an intermediate process in the form of certain polymorphisms in mitochondrial uncoupling proteins which can lead to oxidative stress [103]. Diabetes has become a very important public health concern, and is among the leading causes of mortality and morbidity in developed countries. Although many researches have shown its connection with telomeres and telomerase dysfunction, the current data are still inconclusive.

### 2.3. Autoimmune Diseases

There is a long list of autoimmune diseases like rheumatoid arthritis (RA), systemic lupus erythematosus (SLE), multiple sclerosis, etc. that are characterized by immune system dysfunction and thus inflammatory cascades seem to play a major part in disease onset and progression [104,105]. The mechanisms involving immune system impairment in autoimmunity are numerous and complicated, and while each disease has its specific characteristics, there are certain aspects overlapping between these disorders. For example, cytokines/chemokines and the loss of their homeostatic balance seems to be of extreme importance. CD4 T helper cells and more particularly a deregulation of their differentiation, IL-6, IL-12, IL-1, TNF-a and IFN-γ seem to be major key points in disease severity and evolution [104,105,106].

In RA, telomeres were analyzed in PBMCs, CD4 T cells, CD8 T cells, and hematopoietic progenitor cells. In every case, telomere erosion was higher in patients when compared to healthy controls but this abnormal shortening was not correlated with disease duration or severity [107,108,109]. More particular, many studies agree that RA patients exhibit accelerated telomere shortening but this erosion occurs only in the early years of life and do not continue to shorten as a function of age [110]. In accordance telomerase expression was found to be deregulated in many cases but again no association with disease activity could be identified [108]. For example, Fujii et al. showed that T cells from RA patients exhibit diminished capability of upregulating telomerase activity, while other studies showed that lymphocytes from RA patients show increased telomerase activity [108,110]. It is not clear whether these findings are intrinsic defects of RA or the result of chronic inflammation although many studies suggest that such a connection exists [107,108,109,111,112].

The status is different in SLE where telomere attrition was also found to be higher in many cell types such as leucocytes from patients when compared to normal controls, but the results are contradictory regarding the association to disease activity [113,114,115]. There are groups reporting that premature telomere loss is connected to disease activity where others have reported no connection at all [114,115,116]. One study for example showed increased telomere loss in patients suffering from SLE and a positive correlation between disease activity and anti-telomere antibody levels, as long as a negative correlation of telomere length with Vitamin D levels [116]. On the other hand, telomerase activity is higher in patients and appears to be correlated with disease activity especially in B lymphocytes [117,118,119]. In addition, PBMCs from patients suffering from SLE exhibit different expression motifs in certain telomeric proteins such as TRF1, TRF2, POT1, etc. when compared with healthy individuals, a finding contributing to the notion that telomerase deregulation might be essential to disease pathogenesis [111,114,115,120].

Similarly, telomere length was found to be shorter in T cells from patients with psoriasis or atopic dermatitis whereas telomerase was found to be more active when compared to normal controls, indicating a chronic stimulation of T cells [121]. Accordingly, Liu et al. not only confirmed the above results in psoriatic patients but they also observed an association between telomerase activity and disease severity but not duration [122]. On the other hand, when telomere length was analyzed as a possible predictive marker, it failed to show any association with the development of metabolic syndrome or cardiovascular disease in psoriatic patients [123].

Another serious chronic autoimmune disorder worth mentioning is multiple sclerosis (MS). It has been discovered that telomeres from PBMCs show increased telomere shortening when compared to normal subjects, a phenomenon according to the authors caused by oxidative stress (OS) which in turn can lead to various inflammatory responses. More importantly, the authors continue to suggest that decreased telomere length and increased oxidative stress reflects the severest state of the disease [124].

The possible connection between telomeres/telomerase and inflammation has not yet been extensively studied in many autoimmune disease. However, since there are many studies connecting telomere instability with many inflammatory processes, and since immune system, impairment is at the core of autoimmune diseases, it is possible that an interconnection between telomerase and inflammation exists in these disorders that is yet to be uncovered. For example Tamayo et al. showed shorter telomeres in many rheumatologic pathologies possibly depending on the presence or absence of systemic chronic inflammation. Shorter telomeres were observed in disorders like RA, ankylosing spondylitis and psoriatic arthritis while osteoarthritis, a disorder without a chronic inflammatory component showed no difference in telomeres when compared to age-matched controls [125]. Moreover, in a review by Dehbi et al. about telomeres in rheumatic disorders, the authors conclude that although accelerated telomere shortening might be responsible for disease predisposition, the possible causes for this shortening include genetics, oxidative stress/DNA damage, chronic inflammation and cellular turnover [110].

### 2.4. Renal Failure

There is accumulating evidence the past few years that persistent low grade chronic inflammation plays a major part in disease progression, severity, mortality and morbidity in patients suffering from renal failure or chronic kidney disease (CKD) [126,127]. That is why chronic inflammation in CKD is extensively studied since it has been hypothesized to be a potential therapeutic target [128]. TNF-a, CRP, IL-6, IL-1, IL-23, reactive oxygen species (ROS) and others, have all been implicated in promoting impaired renal function [129]. Impaired immunity/inflammation can be predictors of mortality and morbidity contributing to the risk of CKD and it seems very likely that telomeres/telomerase are very important aspects of this relationship [130,131]. Oxidative stress and chronic inflammation can cause lymphocyte telomere shortening resulting in T-cell dysfunction and ultimately susceptibility to kidney infection and injury. In addition, this persisting immune system activation engages fibroblasts and increases collagen deposition and renal fibrosis [132].

Our lab was among the first to report a decrease in telomerase activity in patients suffering from CKD. Moreover, this activity was lower in patients with intermediate stages of CKD and not in dialysis ones, though the enzyme’s activity was also found to be pathological in dialysis when compared to normal controls. In addition, *hTERT* exhibited low levels of expression in intermediate stages of the disease but not in dialysis patients while our study also confirmed the inflammatory status of these patients [130,131]. Similarly, it has been reported that telomere length is affected from CKD duration when measured in DNA from whole blood samples. The longest telomeres were observed in primary stages of the disease while the shortest in patients with intermediate disease duration and not in long term CKD patients [133]. Similar results were also obtained by a study from Stefanidis et al. where although telomere length and telomerase activity in PBMCs had no differences between patients and healthy individuals, shorter telomeres were associated with the duration of dialysis [134]. In a large study by Raschenberger et al., an association was reported between shorter leucocyte telomere length and CKD progression and this association was found to be stronger in active smokers and diabetics. The authors continue to comment that this is probably due to increased oxidative stress and inflammation [135]. Mice lacking functional telomerase have been shown to exhibit marked reductions in renal function and regeneration 7–30 days after ischemia-reperfusion injury. In other words, shorter telomeres possibly contribute to increased renal injury and decreased recovery after damage [132,136]. Finally, it should be noted that De Vusser et al. demonstrated an association between shorter leucocyte telomere length and arteriosclerosis in smaller intrarenal arteries suggesting a central role of replicative senescence in the progression of renovascular disease [137]. Current data support a connection of telomeres and telomerase in the chronic inflammatory status of renal failure though the exact connection still remains unknown and more studies are needed in order for every molecular aspect of this connection to be uncovered.

### 2.5. Cardiovascular Disease

Atherosclerosis is the dominant cause of cardiovascular disease (CVD) including myocardial infarction (MI), heart failure, stroke and claudication [138,139]. Activated endothelium with the expression of adhesion molecules seem to be early events in atherosclerosis, allowing monocytes, T-cells, dendritic cells, mast cells, neutrophils and B-cells to accumulate to the endothelium [140]. Although endothelial dysfunction is one of the very early events associated with CVD, it is becoming increasingly clear that chronic inflammation and immune system deregulation play important roles in the onset and progression of disease [141,142]. Both in vitro and in vivo studies have shown that IL-1β is a potent pro-inflammatory and atherogenic cytokine with a wide range of biological effects able to stimulate the NF-κB pathway. Other systemic inflammatory markers found to be elevated in CVD are oxidized low-density lipoprotein (oxLDL), CRP, fibrinogen, IL-6, TNF-a and IL-6 [140,141,143].

There have been studies suggesting that telomere attrition contributes to endothelial dysfunction while higher rates of attrition have been reported in aged vessels with increased shear wall stress [144,145]. Leucocyte telomere length has also been reported to be shorter in patients with coronary artery disease while those patients who exhibited shorter than average telomere length had between 2.8 and 3.2 fold higher risk of myocardial infraction [144]. It should also be noted that heart tissue from patients with cardiac hypertrophy exhibit increased telomerase activity but also increased telomere loss [144]. Moreover, telomere biology has also been linked to hypertension since telomere shortening significantly contributes to increased pulse pressure and pulse wave velocity in male patients, while hypertension also in men has been associated with shorter leucocyte telomere length [146]. Intrarenal arteriosclerosis has also been associated with shorter leucocyte telomere length, indicating that replicative senescence plays a central role in the progression of renovascular disease, independent of calendar age [137]. Shorter leucocyte telomere length has also been associated with increased concentrations of certain inflammation markers such as oxLDL, CRP and IL-6 [147]. Interestingly, Gizard et al. discovered that *hTERT* is overexpressed in macrophages isolated from atherosclerotic lesions and when this expression is limited experimentally, a senescent phenotype is observed. In addition, the researchers continued to report that inflammatory mediators such as LDL, LPS and TNF-a induce telomerase activity in macrophages while they also discovered a highly conserved NF-κB response element located at the proximal promoter region of *hTERT.* In other words, it seems in response to inflammatory stimuli NF-κB rapidly recruits at this particular site promoting *hTERT* transcription [148]. It is also worth mentioning that Calvert et al. suggested leucocyte telomere shortening promotes high-risk atherosclerotic plaque subtypes by increasing pro-inflammatory activity [149].

### 2.6. Psychiatric and Neurological Disorders

Psychiatric disorders such as depression, schizophrenia, anxiety disorder, bipolar disorder (BD) and post-traumatic stress disorder (PTSD) pose serious and emerging global health threats and have in recent years been associated with accelerated aging, chronic inflammation and immune system deregulation [150,151,152,153]. Depression is associated with higher levels of IL-1β, IL-6, IFN-γ and TNF-a [154] schizophrenia has been find to exhibit increased concentrations of IFN-γ, IL-2 and IL-6 [153] whereas IL-2, IL-6,IL-8 and IFN-γ were found to be overproduced during BD [152]. The list goes on, while there are also studies connecting psychiatric disorders with telomeres and telomerase. Leucocyte telomere length has been studied in many psychiatric disorders. Shorter telomeres have been observed in depression than healthy controls and in addition shorter telomeres where associated with severity and duration of the disease [151,155]. Studies about telomere length in BD are contradicting and inconclusive but it is interesting to state that lithium therapy seems to be associated with longer telomeres especially in lithium responding patients [151]. Moreover, longer telomeres were reported in schizophrenia but in patients receiving medication whereas shorter telomeres have also been observed in PTSD. In addition, increased telomerase activity has been connected with stressful disorders and surprisingly this activity was associated with shorter telomeres [151]. For example, although acute laboratory stress has been reported to be associated with higher telomerase activity, chronic stress patients exhibit decreased telomerase activity while increased telomerase activity is observed in patients with major depressive disorder [156]. There are not many studies about the relationship between telomeres/telomerase and inflammation in psychiatric disorders. Since chronic inflammation is observed in many of these diseases and inflammation is connected with deregulated telomeric status, it is highly likely that inflammation plays a major part in telomere shortening of these patients or vice versa. For example, it has been discovered that short telomeres in leukocytes from patients with depression are associated inversely with inflammatory markers’ concentration [151,157]. Similar findings were also observed in patients with early life stress [158].

Alzheimer’s disease (AD) is the major cause of dementia in older people affecting more than 40% of individuals over the age of 85 [159]. Collective data suggest that neurons and glia are subject to cellular senescence resulting from accumulative DNA damage, oxidative stress and inflammation factors that increase during brain aging and neurodegenerative disease such as AD [159]. Studies regarding leucocyte telomere length in patients with AD are inconclusive since some studies show shorter telomeres and others are unchanged when compared to healthy controls [159,160]. In addition, it has been reported that telomerase is expressed in neurons and glia protecting the cells from oxidative stress and tau pathology [159,161,162]. Data regarding telomeres and telomerase status are also inconclusive in Parkinson’s disease (PD), another common neurodegenerative disorder where oxidative stress and inflammation have also been implicated in its etiology [163,164]. A meta-analysis by Forero et al. showed no evidence of shorter telomeres in patients suffering from PD while Schürks et al. reported that men with shorter leucocyte telomere length have a surprisingly lower risk of developing PD [165,166]. On the other hand, it must also be noted that shorter telomere length has been observed in white blood cells from PD patients in the presence of high oxidative stress [167]. 

### 2.7. Chronic Infections

Since leucocytes are among the very few cell types of a mature organism to exhibit telomerase activity, the enzyme’s proper regulation inside the host’s defense system should be crucial in handling acute and chronic infections [168]. Telomeres and telomerase biology is being studied in HIV and other viral chronic infections since these patients exhibit signs of chronic inflammation, accelerated aging and premature immune system senescence [168,169,170,171]. HIV in particular is characterized by CD4 T cell depletion by apoptosis and CD8 T cell senescence. This exhaustion observed in immune system cells is probably due to the progressive loss of cell renewal capacity because of the continuous T cell turnover [168,169,170]. There are many studies reporting shorter leucocyte telomere length and reduced telomerase activity in infected individuals and try to link the immunosenescence observed in HIV population with this telomerase dysfunction [169,172,173]. In addition, shorter telomeres and lower telomerase activity has been found to be associated with viremia and with patients exhibiting signs of advanced disease [168,173,174,175]. Moreover, reverse transcriptase inhibitors used in HIV treatment have been shown to also inhibit telomerase activity [176,177]. In other words, though these therapeutic options are effective in HIV therapy, telomerase inhibition might promote immune system dysfunction and other HIV-related comorbidities. For example, mitochondrial accelerated aging observed in these patients has been speculated to be induced by telomerase inhibition caused by anti-retroviral treatment [176,177]. Telomerase activity in CD8 T cells has also been proposed that it could serve as a biomarker of immune system status in HIV infected individuals [169].

Telomerase activity has also been found to be reduced in lymphocytes from patients suffering from hepatitis B virus infection (HBV) while *hTERT* expression was also found to be lower in patients with hepatitis C virus infection (HCV) when compared to normal controls [178,179]. Cytomegalovirus (CMV) is another common chronic viral infection. Patients with higher CMV IgG antibody levels representing increased chronic antigenic stimulation have been reported to be associated with lower PBMC telomerase activity although the data regarding telomere shortening and CMV infection still remain inconclusive [180]. Apart from viral, telomerase has also been found to be associated with bacterial infections as well, such as *Listeria monocytogenes* where this particular pathogen has been shown to induce hTERT degradation, an event crucial to bacterial replication [181]. Although the purpose of this review is not to detail the relationship between telomerase and cancer, great importance must be given to the fact that many viruses with the ability to establish latent human infections cause cancer. It is estimated that human viruses are responsible for one fifth of cancers worldwide and they do so by regulating telomerase transcription in order for cell immortality to be established. Epstein-Barr virus, human papillomavirus, hepatitis B and C are among the many viruses with the ability to cause carcinogenesis by employing different mechanisms in increasing telomerase reverse transcriptase transcription [182].

### 2.8. Lifestyle Habits

Telomerase activity and telomeric length have been associated with many everyday life characteristics prevalent in modern societies. Several studies have linked reduced telomerase activity with several unhealthy lifestyles. For example, chronic everyday stress is associated with decreased telomere length and reduced telomerase activity [183,184]. In addition, higher levels of nocturnal cortisol expression, a hormone associated with chronic stress, is related to shorter telomeres while in vitro the addition of cortisol seems to diminish telomerase activity [183]. Apart from everyday psychology, dietary habits also seem to influence telomere/telomerase homeostasis. Short telomeres and a decrease in telomerase activity have been reported in patients with high body mass index (BMI), higher circulating glucose levels and abdominal fat while a healthy lifestyle with the intake of many antioxidants, fruit/vegetables, less processed meat and more exercise has been linked with longer telomeres [185,186]. Moreover, it has been shown that interventions in stress and eating habits can increase telomerase activity while Mediterranean diet has been associated with high PBMC telomerase activity, longer telomeres and a healthier status in general [183,185,186]. Telomerase activity in persons under the Mediterranean diet exhibits a negative correlation with inflammation markers while a higher dietary fat intake triggers PBMCs to secrete more inflammatory molecules. Although there are a lot of data suggesting a relationship between diet, lifestyle, telomeres/telomerase and inflammation, the details behind this possible connection are still vague [183,186]. Interestingly, studies have also associated poor sleep quality and short sleep duration with shorter leukocyte telomere length [110].

It was stated earlier that high body mass is associated with shorter telomeres. Obesity is a well-known cause for many disorders while it is also considered to be a state of increased inflammation and oxidative stress [108,116]. In addition leptin, a hormone exhibiting high plasma levels in obese subjects has also been shown to act as an inflammatory molecule, triggering IL-6 and other cytokines [112]. It has been suggested that obesity may also increase the risk of telomere shortening. In a meta-analysis by Mundstock et al. a tendency towards demonstrating a negative correlation between obesity and telomere length was shown but the results were not conclusive and several studies showed heterogeneous results [116]. In postmenopausal women, body mass and body fat percentage were inversely associated with telomere length while estrogen levels showed a positive association [110]. Moreover, telomere length was found to be negatively associated with telomere length in leukocytes from adults having gained over 30 kg in body weight when compared to individuals that maintained their weight [108]. Finally, it has been observed that leukocyte telomeric length can be affected by weight loss. Particularly, the more weight someone lost the greater lengthening of telomeres was observed [125,187]. In addition, a weight loss program for obese adolescents showed that longer telomeres at baseline are associated with an improvement in glucose tolerance and inflammation status [116].

Healthy telomerase activity and long telomeres are associated with longevity and as a consequence it is inevitable to hypothesize that every habit contributing to unhealthy lifestyles can possibly affect telomerase levels. In this context, alcohol consumption has been found to be associated with shorter telomere length. More particular, PBMC telomeres were shorter in alcoholics and it correlated inversely with the amount of drinks per day [188]. In addition, telomeres from the esophageal epithelium of alcoholics were shorter than normal controls although no correlation with tissue inflammation could be observed [188,189]. On the other hand, though, it should also be noted that recent studies have uncovered the importance of chronic inflammation in the pathogenesis of many systemic manifestations of alcoholism. It has been shown that cytokines and ROS overproduction in alcoholics induce damage in many organs even in patients without liver damage [190].

### 2.9. Other Disorders

Telomere shortening and reduced telomerase activity with or without an inflammation connection have also been studied in many more pathological contexts than those described in the present study. For example, patients with advanced primary biliary cirrhosis retain significantly less telomerase activity than patients with early stage disease while these subjects also exhibit signs of premature cellular senescence [191]. Ulcerative colitis is a well-known inflammatory disorder were premature telomere shortening has been observed in patients’ colonocytes, a fact that can be possibly associated with increased oxidative stress secondary to inflammation [192]. Also, in chronic periodontitis, patients show shorter leucocyte telomere length while a negative correlation was also observed between shorter telomeres, disease severity and markers of oxidative stress [193]. In addition, telomere shortening has been reported in lens epithelial cells of patients suffering from eye cataract, whereas when myoblasts from sporadic inclusion-body myositis where cultured in vitro accelerated telomere shortening and signs of premature senescence were reported [57,194].

## 3. Conclusions and Future Prospects

Many chronic conditions in humans are associated with chronic inflammation, immune system impairment and accelerated aging. In addition, abnormalities in telomere/telomerase system of these patients have been reported in many of these disorders. Since telomerase, an enzyme directly associated with aging, is inactive in most cell types in a mature organism and active in immune system cells, one can easily hypothesize that the immune system dysfunction/accelerated aging observed in chronic conditions is connected with telomeres and telomerase biology. Indeed, a connection of this nature seems to exist since shortened telomeres, observed in aged cells, cause an inflammatory cascade whereas, at the same time, NF-κB, a master regulator of inflammation, seems to directly induce telomerase transcription as stated above. Moreover, many researchers documented correlations between lower telomerase activity and/or shorter telomeres in immune system cells and elevated cytokines in blood serum from patients with chronic disorders. One should also bear in mind that, although aging is a multifactorial and complex procedure, healthy aging and longevity are believed to be associated with longer telomeres and lower inflammation profiles among older individuals [28,195,196]. Despite all of the above, and despite the accumulating data of a strong interconnection between telomerase regulation/activity and inflammation, the mechanistical details and the molecular pathways of this connection have not been uncovered yet.

Due to the emergence of chronic inflammation as a prominent risk factor of many human disorders, researchers hypothesize that harnessing inflammation can have many beneficiary and longevity effects. Interestingly, many of the interventions used so far to regulate and diminish inflammation also seem to positively affect telomere biology as well. For example, inhibition of one of the major cytokines produced by senescent cells, TNF-a, increases telomerase activity and proliferative potential [53]. Another finding worth noting is the pleiotropic effects of statins and angiotensin converting enzyme inhibitors (ACEIs). Statins are lipid lowering drugs and ACEIs are anti-hypertension drugs; both have been used effectively in many patient categories for decades. Both drugs are also considered anti-inflammatory agents as it has been observed to lower circulating cytokines in patients’ blood serum [197,198]. Additionally, both agents have also been found to promote telomerase activity and/or *hTERT* gene transcription [28,199,200,201,202]. It is also worth noting that in certain disorders such as CKD, the possibility of targeted anti-inflammatory therapy is studied extensively with interleukin receptor antagonists and other agents like pentoxifylline already showing promising results in systematically lowering the inflammatory profile of these patients [203]. Lifestyle habits and interventions are also another important factor that needs to be taken into account. A healthy diet, frequent exercise and a low everyday stress profile has been associated with a healthier inflammatory status as many studies have shown [25,204,205]. As stated above, the same lifestyle factors are also connected with longer telomeres and/or higher telomerase activity while psychiatric disorders, obesity, etc. are associated with deregulated telomere/telomerase physiology [22,28,183,201,204,205,206]. It should also be noted that meditation has been reported to reduce stress, downregulate inflammatory genes and increase telomerase activity by up to 43% [156,207], while older married adults with high income are associated with longer telomeres [208].

Novel therapeutic approaches are focusing on activating telomerase for treating age-associated pathologies and immune-compromised chronic disorders. Several telomerase activators have been recently starting to emerge as possible treatment agents like resveratrol, genistein and cycloastragenol [209,210]. The latter is the most well documented one since cycloastragenol was shown to increase telomerase activity, reduce telomere shortening, upregulated *hTERT* and, in general, increased proliferative potential [209,210,211]. In addition, cycloastragenol enhanced immune function of T cells, reducing the percentage of senescent cytotoxic T cells while no adverse effects were observed. It should be stated though that telomerase activation needs to be studied rigorously since uncontrolled activity of the enzyme might as well have cancerous effects [20,209,210]. Moreover, treatment with danazol has recently been shown to promote telomere elongation in PBMCs from patients with telomeric diseases such as bone marrow failure [212].

Most chronic human disorders are associated with immune system dysfunction, i.e., chronic inflammation, a phenomenon associated with abnormal telomere/telomerase physiology in the same cell populations. Though there are many studies suggesting and in some cases proving the interconnecting relationship between immune system aging, inflammation, telomerase dysfunction and disease, still the intermediate details of these connections are unknown. The purpose of this study was to review the scientific literature and document the recent findings regarding telomeres/telomerase biology in chronic inflammatory diseases. Though we are still far from applying current knowledge in daily therapeutic protocols, many studies seem to agree that a combination of exercise, healthy diet, low everyday stress and anti-inflammatory agents’ intake may prove beneficial in promoting human longevity and slowing down the effects of many chronic disorders. The present knowledge in this direction is very poor and further research is certainly needed in order to uncover the true molecular relationships involved in the above phenomena. Only when the molecular roads are completely uncovered, scientists might be able to harness the deleterious effects of chronic inflammation in order to promote human life span.
